# A Multibiomarker Approach to Assess the Health State of Coastal Ecosystem Receiving Desalination Plants in Agadir Bay, Morocco

**DOI:** 10.1155/2019/5875027

**Published:** 2019-12-16

**Authors:** Ahmed Elazzaoui, Abdellatif Moukrim, Latifa Lefrere

**Affiliations:** Laboratory of Aquatic Systems: Marine and Continental Ecosystems, Department of Biology, Faculty of Sciences, Ibn Zohr University, BP 8106, Agadir, Morocco

## Abstract

The present study aims to evaluate the initial health status of two stations receiving seawater desalination plants in Agadir Bay (*Tifnit-Douira* and *Cap Ghir*) and to assess their potential environmental impact on the marine ecosystem health. Six pairs of mussels (*Mytilus galloprovincialis*) were collected at six sampling sites on a monthly basis over two years. Each pair was homogenized to obtain the postmitochondrial fractions (S9). Toxicological effects were measured using a multibiomarker approach based on either acetylcholinesterase (AChE), glutathione S-transferase (GST), catalase (CAT), and malondialdehyde (MDA) rate. The results show a seasonal variation of the biomarkers: their activities increase in summer and decrease in spring and winter. High activities were recorded during summer in Cap Ghir (17.94 ± 0.88; 5.91 ± 052 nmol/min/mg of protein) for CAT and MDA, respectively. In Tifnit-Douira, low activities were recorded during winter for GST (3.74 ± 0.52 nmol/min/mg of protein) and during spring for the CAT (3.52 ± 0.45 nmol/min/mg of protein). The fluctuations in the activities of measured biomarkers could be attributed to different factors including the changes in environmental parameters, the influence of seasonal variation, and the contamination of the aquatic ecosystem. The data obtained in this study should be taken into account in the monitoring and management of the health of the ecosystems when the desalination plants are established.

## 1. Introduction

The national and regional sectoral strategy makers (Green Morocco Plan, Plan Azur, Halieutis, etc.) were planned to realize many development programs and projects in the Souss-Massa region [1–5]. However, water availability is a limiting factor for the implementation of these projects and for any regional development [6, 7], particularly in regions that are characterized by dry climate conditions and overexploitation of water resources (dam and groundwater).

To overcome these problems, the regional council, as a part of its strategy, has invested in the installation of seawater desalination plants, in Agadir Ida Outanane (Cap Ghir) and Chtouka Ait Baha Province (Tifnit-Douira), to produce water and to meet the requirements of development projects [8, 9].

Besides the fact that scientific data of Tifnit-Douira and those of Cap Ghir are not available, the importance of ecological coastal ecosystems and the potential impact of the desalination plants prompted us to launch a new study to assess the initial health status of the ecosystems.

This study was established by using a multidisciplinary approach to assess the coastal ecosystem health by several environmental monitoring programs such as Mussel Watch Program [10–13]. It will be useful to follow the environmental changes of these ecosystems after the implementation of projects. Our research laboratory has often used this approach for several years to evaluate and to follow-up the environmental quality of Agadir Bay ecosystems [14–22]. It is based on the use of many biomarkers, such as acetylcholinesterase, catalase, glutathione S-transferase, and malondialdehyde, as pollution indicators of seawater quality in the sentinel species (*Mytilus galloprovincialis*, *Perna perna*, *Donax trunculus*, *Nereis diversicolor*, *Cerastoderma edule*, etc.).

The aims of this work are to study the initial health state of the coast (Tifnit-Douira and Cap Ghir) receiving desalination plants of the seawater in Agadir Bay and to evaluate the potential environmental impact of the installation of these plants on the coastal ecosystem. Our study combined classical research using biodiversity, population structure, and analysis of water and sediment parameters.

## 2. Materials and Methods

### 2.1. Studied Areas and Sampling Sites

**Figure 1:**
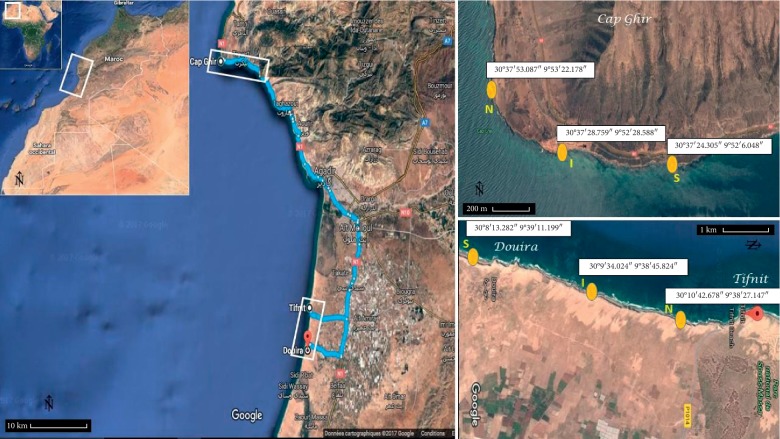
Map of the sampled sites in the coastline: Cap Ghir in the north and Tifnit-Douira in the south of Agadir (Morocco) (South (S), Instal (I), and North (N)), and the distance between the sites is >1 km.

Our investigations were conducted in two stations (Cap Ghir and Tifnit-Douira), with three sampling sites for each station (South, Instal, and North). The first one (Cap Ghir) is located 50 km north of Agadir, and it is characterized by ecological and socioeconomic importance due to upwelling phenomenon [23–25]. The second one (Tifnit-Douira) is located 60 km south of Agadir Bay in the center of Souss Massa National Park (Figure 1).

The choice of these sampling stations and sites was based on their implementation near the desalination plants and also because of the north-south direction of the marine currents [20, 26]. In fact, the southern sites (according to the currents) will receive more environmental impact than Instal and North sites.

### 2.2. Specimen Sampling

Our study was conducted on samples of standardized mollusks with the same shell length (40 to 50 mm) of *Mytilus galloprovincialis*. They were collected from February 2015 to March 2017, quickly transferred to laboratory, and stored immediately at −80°C until use.

The choice of this mollusk was based on many reasons: it is a good sentinel species with sedentary lifestyle, it can be collected easily, and it accumulates pollutants by filtration. Moreover, it has often been used as a sentinel species in such studies, particularly in the Mussel Watch Program [27].

### 2.3. Preparation of Postmitochondrial Fraction

All procedures and preparations were conducted at 4°C. Mussels were thawed, dissected, and drawn out of the shells; the whole soft tissues of each two individual mussels (pool of six pairs) were homogenized for 1 min in a ULTRA-TURRAXT25 homogenizer at 9500 rpm with 1:3 w/v of 100 mM Tris buffer (pH 7.4). The homogenates were then centrifuged for 30 min at 9000*g* (Sigma, 3–16k), and the postmitochondrial supernatant S9 fraction was stored at −80°C until use. All biochemical parameters are measured in this S9 fraction.

### 2.4. Biochemical Analyses

#### 2.4.1. Acetylcholinesterase Activity

The AChE activity was measured by the method described by Ellman et al. [28] with the use of acetylthiocholine iodide as the substrate. The enzyme kinetic measurements were performed at 412 nm every 15 seconds for 2 min at 25°C. The enzyme activity was expressed as nanomoles of acetylthiocholine/min/mg of S9 protein.

#### 2.4.2. Glutathione S-Transferase Activity

The GST activity was determined by the method described by Habig et al. [29] using glutathione (GSH) and 1-chloro-2-4-dinitrobenzene (CDNB) as the substrate. Enzyme activity was estimated by monitoring changes in the optical density (ΔOD/min) at 340 nm for 2 min at 25°C. The GST activity was expressed as nmol/min/mg of protein.

#### 2.4.3. Catalase Activity

The CAT activity was measured according to the method of Aebi [30] in which the decrease in absorbance at 240 nm caused by the consumption of hydrogen peroxide H_2_O_2_ was quantified. The activity of CAT was expressed as nmol/min/mg of protein S9.

#### 2.4.4. Malondialdehyde Rate

The determination of MDA contents was estimated by the thiobarbituric acid, according to the method described by Sunderman et al. [31] using 1,1,3,3-tetraethyloxypropane as the standard. The absorbance was measured at 532 nm, and MDA content was expressed as nmol/mg of proteins.

#### 2.4.5. Protein Assay

Total protein concentration in the S9 fraction was determined as stated by Lowry et al. [32] using bovine serum albumin as the reference.

### 2.5. Statistical Analysis

Data were expressed as mean ± standard deviation (m ± SD) and repeated in six replicates. Comparison of the means was done by the analysis of variance (ANOVA). The statistical significance of the differences between samples was determined using Statistica (version 6) software and Fisher's least significant difference (LSD) test. A *p* value less than 0.05 was considered statistically significant.

## 3. Results

### 3.1. Biomarkers Response in Cap Ghir Station

**Figure 2:**
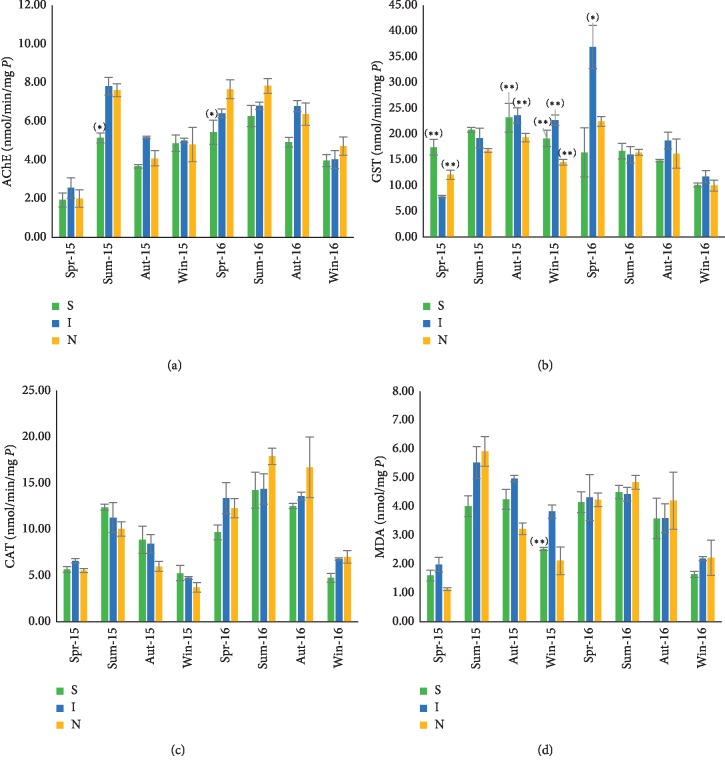
Seasonal variations of acetylcholinesterase (a), glutathione-S-transferase, (b), catalase activity, (c) and malondialdehyde level (d) in *Mytilus galloprovincialis* collected from 3 sites (South (S), Instal (I), and North (N)) of the coastline Cap Ghir (Spr: spring; Sum: summer; Aut: autumn; Win: winter). Data were expressed as mean ± SD (*n*=6). Values indicate significant difference between sites (^*∗*^) and between stations (^*∗∗*^), *p* < 0.05.

The seasonal variation of biomarkers in *Mytilus galloprovincialis* from three sites of Cap Ghir station is illustrated in Figure 2. The results showed that the AChE activity (Figure 2(a)) displays the same tendency of seasonal variations in the three sites of Cap Ghir station. The highest value was observed during summer (7.87 ± 0.38 nmol/min/mg of protein in 2016), and the lowest one was recorded in spring (1.94 ± 0.37 nmol/min/mg of protein in 2015). The statistical test regarding the three sites does not show any significant difference, except in summer 2015 and spring 2016 using AChE biomarkers.

Figure 2(b) shows the seasonal variations of GST in the three sites of Cap Ghir station. It reveals that this biochemical parameter has similar seasonal variations for different sites. High values were measured in summer, and low values in spring and winter. However, we recorded the highest values in spring 2016 (36.91 ± 4.19 nmol/min/mg of protein) and the lowest one in spring 2015 (7.86 ± 0.23 nmol/min/mg of protein).

A comparison between the sites for the same seasons did not show any significant difference, during the two years, excluding the Instal site during spring 2016.

A similar tendency of seasonal variations was observed for the CAT activity (Figure 2(c)): high activity in summer 2016 (17.94 ± 0.88 nmol/min/mg of proteins) and weak activity in winter 2015 (3.74 ± 0.52 nmol/min/mg of proteins).

The statistical analysis of CAT in the three sites of Cap Ghir station did not show any significant difference during the studied period. However, the results show that, in Cap Ghir, the CAT induction was more significant in 2016 compared to 2015.

For the MDA rate (Figure 2(d)), we recorded the maximum value in summer 2015 (5.91 ± 0.52 nmol/mg of proteins) and the minimum one in spring 2015 (1.13 ± 0.04 nmol/mg of protein). The statistical test did not show any significant difference between the three sites in Tifnit-Douira and Cap Ghir.

### 3.2. Biomarkers Response in Tifnit-Douira Station

**Figure 3:**
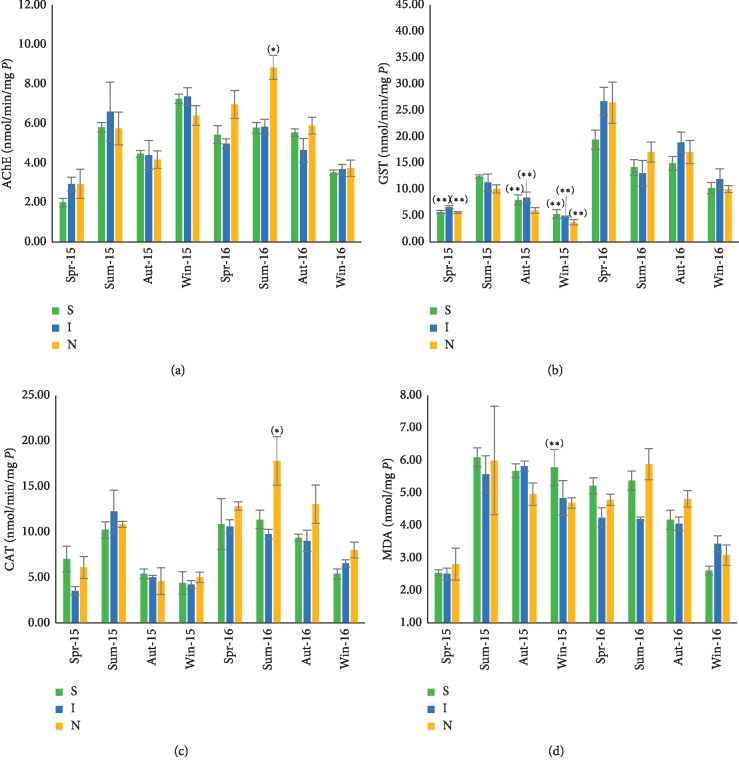
Seasonal variations of acetylcholinesterase (a), glutathione S-transferase, and (b), catalase activities (c) and malondialdehyde level (d) in *Mytilus galloprovincialis* collected in 3 sites (South (S), Instal (I), and North (N)) of the coastline Tifnit-Douira (Spr: Spring; Sum: Summer; Aut: Autumn; Win: Winter). Data were expressed as (mean ± SD) (*n*=6). Values indicate significant difference between sites (^*∗*^) and between stations (^*∗∗*^), *p* < 0.05.

The AChE activity was high in summer (8.85 ± 0.61 nmol/min/mg of protein in 2016) and low in spring (1.99 ± 0.37 nmol/min/mg of protein in 2015) (Figure 3(a)). Similar seasonal variations were recorded in the three sites of Tifnit-Douira station. The only significant differences were recorded in the North site of Tifnit-Douira during the summer of 2016.

The results obtained for the GST activity in Tifnit-Douira (Figure 3(b)) showed that the maximum of this activity has been registered in spring-summer (26.75 ± 2.66 nmol/min/mg of proteins for 2016), and the minimum values were recorded in winter (3.74 ± 0.52 nmol/min/mg of protein for 2015).

The seasonal variations show a similar tendency in the three sites. No significant difference was observed between the sites during the same season and during the two years.

The CAT variation reveals a seasonal profile, with an increase in summer 2016 (17.71 ± 2.65 nmol/min/mg of protein) and a decrease (3.52 ± 0.45 nmol/min/mg of protein) in spring 2015 (Figure 3(c)). The statistical tests between sites show the same values in every season except summer 2016.

The study of the MDA rate during the study period (Figure 3(d)) showed a seasonal trend for this station with a maximum value in summer (5.83 ± 0.33 nmol/min/mg of proteins in 2015) and a minimum value in spring (1.73 ± 0.21 nmol/min/mg of protein for 2015). The statistical test did not show any significant difference between sites of Tifnit-Douira station.

## 4. Comparison of Biomarkers Response in Cap Ghir and Tifnit-Douira Stations

Even though the three sites of the two stations and for all biomarkers showed a seasonal tendency with similar profile and variations, the comparison between biomarkers values obtained for each season in stations Cap Ghir and Tifnit-Douira during 2015 and 2016, the AChE activity did not show any significant difference.

The GST activity shows a significant difference between stations during spring, autumn, and winter of 2015, while no difference was observed in 2016.

In 2016, we noticed an excessive induction of GST activity compared to 2015 especially in Instal site of Cap Ghir during spring in 2016. The measured values were much higher during these two years in Cap Ghir compared to the Tifnit-Douira, which presents the same range during the studied period.

For the CAT activity, the test does not show a significant difference, but we recorded induction of CAT during the 2016 seasons compared to 2015. While for the MDA, the only significant difference was recorded in the southern site during winter 2015.

## 5. Discussion

The AChE activity has a seasonal profile, i.e., higher activity in summer and lower one in spring. The difference in AChE activity is noted in the southern sites during the summer of 2015 and spring of 2016 in Cap Ghir and during the summer of 2016 in the North site of Tifnit-Douira. Such a difference is probably related to the change in environmental conditions. The measured values in *Mytilus galloprovincialis* during the spring of 2015 show that this biomarker of neurotoxicity was significantly inhibited in mussel populations in these two stations. This inhibition is actually attributed either to the presence of contaminants in the environment, physiological change state, environmental parameters, or to the reproductive cycle of mussels.

The results of the seasonal variations of AChE activity in the present study are in concordance with those reported by Abbassi et al. [22], using the same mollusk as a sentinel species in the coastline of Sidi Ifni (South of Morocco). AChE activity is inhibited by the contaminants such as carbamate and organophosphate pesticides used in agriculture [19]. This inhibition was also observed in other species of polychaete *Nereis diversicolor* [17] and in *Diopatra neapolitana* [33]. Other authors associate this activity in mussels to the reproductive cycle [34]; however, the maximum of AChE activity coincides with spawning period, and the minimum one overlapped with the sexual rest [35, 36].

The contamination by heavy metals can also cause AChE inhibition during spring. Moukrim et al. [37] reported that the highest level of contamination by Cd, Cu, and Zn was observed in winter and spring, and the lowest one was in summer and fall in Agadir Bay. This contamination is imported by seawater mass of currents upwelling, which took place in these sites' coasts. Similar results are reported by Banaoui et al. [38] and De Lima et al. [39]. Nadir et al. [21] reported that Mercury contamination has a direct influence on biomarkers in *Donax trunculus*. Other types of pollution can affect AChE activity, like wastewater [22], contamination by polycyclic aromatic hydrocarbons (PAHs), and organic chemicals [14, 40–42]. Our results corroborate those of Lagbouri [43] and Moukrim et al. [16] in *Anza* beach (Agadir Bay) using the other sentinel species *Donax trunculus*. They measure higher value in summer and lower one in spring in the polluted site.

The lowest AChE activity in soft mass of organisms in the South site of Cap Ghir could be due to the influence of hydrodynamics. The same activity was observed in Tifnit-Douira because of the very important dose of fertilizers and pesticides that arrived to the sea by drainage of Oued Souss and Oued Massa; they are then sprinkled by the marine currents (direction North to South) and they affect organisms, especially in spring, which overlaps with maximum inhibition of AChE enzyme activity.

The organochlorine pesticide (OCP) concentration is high in the sediment and in the sentinel species *Nereis diversicolor* during spring in Oued Souss estuary according to Agnaou et al. [19]. Banaoui et al. [20] showed that the AChE activity revealed a significant inhibition in three mollusk species (*D. trunculus*, *P. perna*, and *M. galloprovincialis*) following a contamination by organophosphorus and organochlorinated pesticides. The reported decrease in the AChE activity in Tifnit-Douira station may also be related to the presence of sand dunes in the coastal zone that could be brought into the sea by downwind transport in the region that import sprayed pesticides in the agricultural zone accumulated in the sediment. The presence of the army field in Tifnit-Douira may have some consequences; the metals and chemical residues of ammunition can reach the sea by sand movements and cause inhibition of AChE activity.

The results obtained in this work showed that GST activity varies following a seasonal tendency, with high values during summer and lowest ones during spring and winter. At the same time, we have registered a significant difference between stations for GST activity during spring, autumn, and winter of 2015. This kind of variations could only be due to the change in the environmental conditions during the seasons of the studied period. The peak of GST activity and the significant difference between sites was registered in the Instal site of Cap Ghir during spring 2016 compared to Tifnit-Douira station. This stimulation in the two stations could be explained by the overlap of sampling with a period of high precipitations during 2016. They can bring different types of pollutants to the sea by Oued Souss and Oued Massa. Those pollutions contribute to the stimulations of the detoxification processes. The GST is the second enzyme phase involved in the metabolism of the detoxification of lipophilic organic contaminants [44, 45]. The apparent increase in the GST activity may be attributed to the presence of different types of pollution in aquatic ecosystems, which stimulates the cellular detoxification process of xenobiotics [44–46]. It is also considered like an adaptive response to altered environment reported by Abbassi et al. [22]. Many other studies have described a similar relationship between environmental pollution and GST activity in mussels and other sentinel organisms in marine ecosystems.

High GST activity levels are caused by exposing molluscs to pesticides [20], heavy metal pollutants [21, 47], wastewater [15–18], and some other toxins such as polycyclic aromatic hydrocarbons (PAHs) and polychlorobiphenyls (PCBs) [48]. The same results of seasonal evolution of GST activity were already obtained by many studies in Moroccan coasts and Estuary (Agadir Bay), like in *Mytilus galloprovincialis* and *Perna perna* [15], in *Donax trunculus* [20, 21], in *Nereis diversicolor* [17], in *Scrobicularia plana* and *Cerastoderma edule* [18]. In addition, the CAT activity shows the same seasonal tendency with an increase in summer and a decrease in spring and winter.

No significant difference was observed between stations and between sites during 2015 and 2016 seasons except for the northern site of Tifnit-Douira during the summer of 2016. When the maximum was observed during the two years of the study, the induction would be attributed to the change in the seawater temperature in the summer and probably to the presence of the same oxidative stress factors like environmental and physiological changes.

The CAT activity is considered as the first line of defense against oxidative stress of organisms [49]. The seasonal tendency of CAT activity obtained in our study confirms the results of *Mytilus galloprovincialis* living in the coast of Sidi Ifni, South Morocco [22], and in other sentinel species like *Donax trunculus* from Anza and Aghroud beaches (Agadir Bay) [16]. Authors reported high CAT levels especially in impacted sites [18, 43], and others confirmed a positive correlation between inductions of CAT activity and some heavy metal concentrations [21].

MDA levels showed high values during all periods of our study, and the only significant difference was registered in the South site comparing the two stations. The same results were revealed by many studies using *Donax trunculus* in Agadir Bay [21], Tunis Golf [50], and Mirleft and Sidi Ifni coast line (South of Morocco) in *Mytilus galloprovincialis* [22]. Such variations have also been reported by El Jourmi et al. [51] in *Perna perna* along the coast of the big Casablanca. Several other studies have described that MDA levels may be positively correlated with the level of certain pollutants such as heavy metals and organic compounds [52, 53]. Bendjoudi et al. [54] reported that biological contamination by *Staphylococcus aureus* causes induction of the GST, CAT, and MDA.

The results show that the investigated biomarkers present the same tendency: increase in summer and decrease in spring and winter excluding the spring of 2016. We observed high stimulation of GST, CAT, and MDA, accompanied by the perturbation in environmental parameters (*T*, pH, salinity, etc.) in seawater due to high pluviometry registered in this period.

## 6. Conclusion

The results obtained during our investigations contribute to the assessment of the initial health status of the sites that will receive the two desalination plants of seawater in Agadir Bay.

The use of biochemical parameters to assess the health status of both studied ecosystems showed that these parameters present variations that can be attributed to natural factors, while others may be affected due to the presence of contaminants in the environment.

This study will be of great use for the monitoring and management of the two ecosystems during and after the installation of the two seawater desalination plants. Furthermore, it will minimize the impact as much as possible in these two stable ecosystems.

## Data Availability

The data used to support the findings of this study are available from the corresponding author upon request.
